# Deep Learning Methods for Wood Composites Failure Predication

**DOI:** 10.3390/polym15020295

**Published:** 2023-01-06

**Authors:** Bin Yang, Xinfeng Wu, Jingxin Hao, Tuoyu Liu, Lisheng Xie, Panpan Liu, Jinghao Li

**Affiliations:** 1 College of Material Science and Engineering, Central South University of Forestry and Technology, Changsha 410004, China; 2 College of Furniture and Art Design, Central South University of Forestry and Technology, Changsha 410004, China; 3 Department of Energy, Environmental, and Chemical Engineering, Washington University in St. Louis, Saint Louis, MO 63130, USA

**Keywords:** deep learning, glulam, wood failure percentage, measurement

## Abstract

For glulam bonding performance assessment, the traditional method of manually measuring the wood failure percentage (WFP) is insufficient. In this paper, we developed a rapid assessment approach to predicate the WFP based on deep-learning (DL) techniques. bamboo/Larch laminated wood composites bonded with either phenolic resin (PF) or methylene diphenyl diisocyanate (MDI) were used for this sample analysis. Scanning of bamboo/larch laminated wood composites that have completed shear failure tests using an electronic scanner allows a digital image of the failure surface to be obtained, and this image is used in the training process of a deep convolutional neural networks (DCNNs).The result shows that the DL technique can predict the accurately localized failures of wood composites. The findings further indicate that the UNet model has the highest values of MIou, Accuracy, and F1 with 98.87%, 97.13%, and 94.88, respectively, compared to the values predicted by the PSPNet and DeepLab_v3+ models for wood composite failure predication. In addition, the test conditions of the materials, adhesives, and loadings affect the predication accuracy, and the optimal conditions were identified. The predicted value from training images assessed by DL techniques with the optimal conditions is 4.3%, which is the same as the experimental value measured through the traditional manual method. Overall, this advanced DL method could significantly facilitate the quality identification process of the wood composites, particularly in terms of measurement accuracy, speed, and stability, through the UNet model.

## 1. Introduction

Wood failure percentage (WFP) is one of the important bases for evaluating gluing performance and plays an important role in assessing the quality of glulam products [1]. However, the traditional WFP measurement method is performed by hand, which introduces a wide margin for error and a great deal of randomness [2,3,4], and cannot adapt to the current rapid development of the production of glulam products. Therefore, a quick, accurate, and automated technique for measuring WFP is required.

To be able to explore an automated method of measuring the WFP, some scholars have explored several measurement methods [5]. C. Frihart et al. [6] conducted measurements by laser scanning a profilometer to investigate the effect of the wood failure level on the measurement results. The experiments showed that the method has good measurement results. Still, the adhesive color change has a greater impact on the measurement accuracy of the method. Oliver Klaüsler et al. [7] performed measurements with the help of UV images [8] of the fracture surface, but the method is still a human-driven measurement method that allows for large measurement errors. Aicher Simon et al. [9] proposed a threshold segmentation method based on a Bayesian matting algorithm for wood failure surface image (WFSI) with large differences in color between adhesive and wood so that the wood failure areas became white and the background areas became black, and the WFP was automatically measured. However, the mechanical and rigid algorithm will cause large measurement errors in the face of complex adhesive color changes and wood failure level changes. Based on this, Adefemi et al. [10] measured specimens prepared from three adhesives using Fiji-ImageJ [11] software assisted (F-IMJ-A) by manual selection. The results of this method are in high agreement with the ASTM D5266: 2005 and exhibits the advantages of high measurement accuracy [12] and mobility. However, when there are many tests, the measurement error increases as the fatigue of the experimenter increases, making it challenging to apply in engineering practice [13].

Deep learning (DL) has emerged in various fields such as computer vision, speech recognition, and natural language processing [14,15]. The DL process is actually a parameter optimization problem; by defining the loss function as the criterion of network convergence, the network continuously learns the characteristics of the provided training data, and finally, the learned model can be used to solve the problems of prediction and classification in practical engineering [16]. The deep convolutional neural networks (DCNN) is one of the representative models of DL. DCNNs have pushed the performance of computer vision systems to soaring heights on a broad array of high-level problems, including image classification [17] and object detection [18]. Yutaka et al. [19] measured the feature-length of scanning electron microscope images of semiconductor device cross sections by DL. Compared with manual measurement by experts, the feasibility of DL measurement was demonstrated, and the measurement speed was 240 times faster than that of manual measurement. However, the measurement accuracy was affected by the fact that their data set consisted of only 90 images. Tao et al. [20].used three-dimensional (3D) reconstruction and DL methods to detect the air voids in hardened concrete surfaces. The results show that the model results and characteristics greatly influence the measurement results. 

Currently, DL is also applied to wood science, which is mainly limited to two aspects of wood defect (i.e., knots, insect eyes, and cracks) recognition [21] and tree species identification [22]. However, no relevant studies using DL methods to measure WFP have been found. Thus, it is natural to expect that time-consuming and laborious tasks, such as wood failure area measurement in WFSI, can be fully automated by using DL. WFP measurement is different from the above defect recognition; defect recognition belongs to the object detection category, while WFP measurement belongs to the semantic segmentation category. The classical models of semantic segmentation mainly include UNet, FCN, SegNet, PSPNet, DeepLab, etc., and the accuracy of semantic segmentation is related to the receptive fields of the semantic segmentation model, among which, the form of UNet, FCN, and SegNet models to increase receptive fields is similar, but the advantages of Unet in small data sets are more prominent.

Therefore, we propose an efficient and automatic measurement method based on convolutional neural networks. Firstly, we select three semantic segmentation models, U-Net, PSPNet, and DeepLab_v3+, and compare the convergence and prediction results of the three semantic segmentation models. Secondly, the optimal semantic segmentation model is selected to evaluate the feasibility of our method in terms of convergence, measurement results, measurement speed, and measurement stability, which is obtained by comparing it with manual methods. This study elaborates on the mechanism of the semantic segmentation model for measuring WFP and reveals the influence law of the test conditions on the measurement method to provide implications for WFP measurement technology.

## 2. Materials and Methods

### 2.1. Image Acquisition

**Figure 1:**
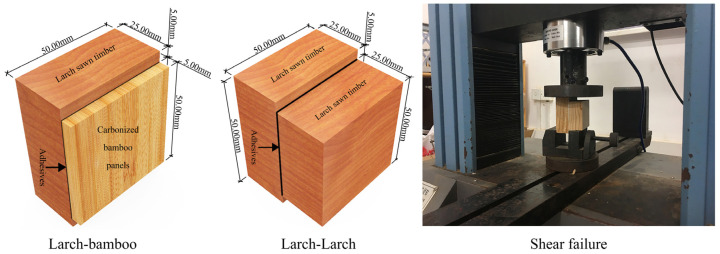
Specimen type and shear damage mode.

Larch (*Larix gmelinii (Rupr.) Kuzen*) sawn timber has an air-dry density of 0.64 g/cm^3^ and measures L × W × H = 2000 mm × 132 mm × 53 mm, without any physical or chemical treatment. The carbonized bamboo boards were purchased from Taobao stores. The preparation process for the carbonized bamboo board is to put the moso bamboo (*Phyllostachys edulis* (*Carriere*) *J. Houzeau*) pieces in boiling water for 12 h, and then put the cooked bamboo pieces in the carbonization furnace for carbonization treatment (time is 90 min, air pressure is about 1 MPa, temperature is 200–300 °C), and the final size is L × W × H = 2000 mm × 600 mm × 5 mm. The PF adhesive purchased from CIMC New Material Technology Co., Hunan, China. was a dark brown liquid with a viscosity of 2000–3000 mPas/25 °C. The MDI, which is a milky white viscous liquid with a viscosity of 150–400 mPas/25 °C, was purchased from Dong Ying Sheng Ji Environmental Protection Engineering Co., Shandong, China. The specimen factors and levels were designed according to ASTM D5266-99:2005 [23] (Table 1), and the specimens (Figure 1) were prepared according to the test combination design shown in Table 2. The PF adhesive curing time was 20 min, the hot-pressing temperature was 135 °C, and the pressure was 1 MPa. The MDI curing pressure was the same as the PF adhesive, and the cold pressing was set at room temperature for 60 min. In contrast, the wet state shear conditions were such that the specimens were immersed in room temperature water at 10–25 °C for 24 h and then immediately subjected to shear failure with a loading rate of 0.038–1.27 cm/min.

**Figure 2:**
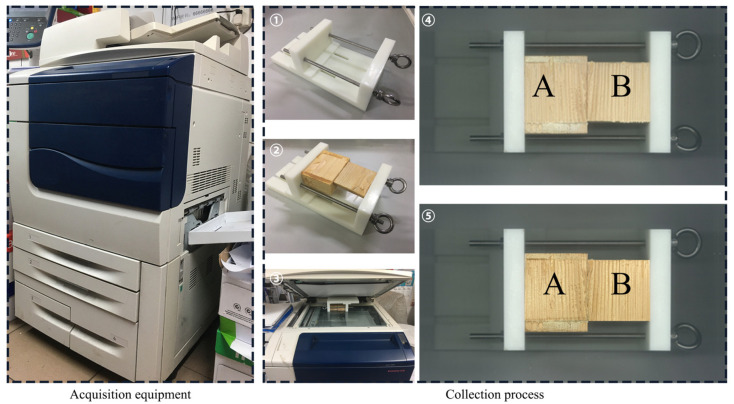
WFSI acquisition equipment and process: ①: Fixture; ②: The specimen is placed in the fixture with the two glued damaged sides up; ③: Place the fixture on the table of the scanning instrument; ④: Acquired scanned image; ⑤: After color correction; A: Specimen length of 60 mm side of the gluing damage surface; B: Specimen length of 60 mm side of the gluing damage surface.

The image acquisition method for this test was based on the requirements of ASTM D5266:2005, and an electronic desktop scanner (FUJI XEROX Color 560) was used to scan the images of the shear failure specimens (Figure 2). After scanning both, a color (RGB) WFSI with a resolution of 400 ppi was obtained and then color corrected by Photoshop CC 2017. Our method has the advantages of uniform illumination, reduced perspective distortion due to changes in distance between the lens and the specimen, low cost, and ease of operation.

### 2.2. Datasets

**Figure 3:**
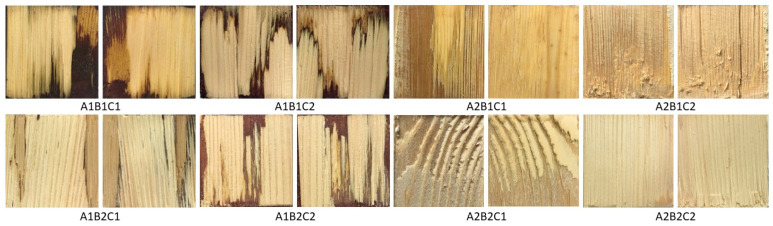
Dataset image type.

The WFSI was acquired for the purpose of this experiment, and we prepared all specimens of the same type ourselves. A total of 320 images were acquired for the initial dataset, and 318 images were finally obtained by screening. Eight image types were included, as shown in Figure 3.

**Figure 4:**
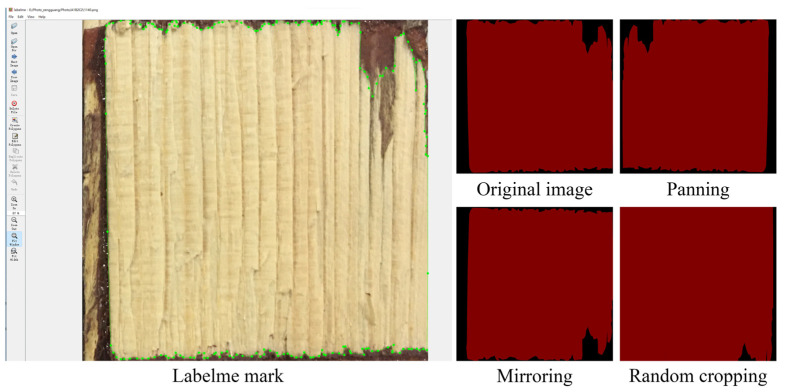
Image augmentation technology.

To improve the training efficiency and prevent the overfitting phenomena, as well as considering that the wood failure areas are the key factors for the accurate measurement of its occupancy, the wrong enhancement effect would change the wood failure areas and cause measurement error. This study uses image augmentation technology (Figure 4) to expand the dataset [24,25]. The total number of expanded labels was 1272. The dataset was divided into a training set, a validation set, and a test set in the ratio of 9:1:1, respectively. The image data used between the three groups did not overlap. The pixels in the images were divided into wood-failure area pixels and non-wood-failure area pixels (both background areas) according to the binarization classification problem, where wood-failure area pixels were labeled in red (RGB (128, 0, 0)) and non-wood-failure area pixels were marked in black (RGB (0, 0, 0)).

### 2.3. Methods

The DL-based semantic segmentation network is a transformation of the current mainstream classification networks into a fully convolutional neural network. The architecture combines semantic information from deep and coarse layers with appearance information from shallow and fine layers to produce accurate and detailed segmentation, which greatly improves the segmentation efficiency. In this study, each pixel point of the original WFSI is classified, and a network model trains the classified image. After training, the WFSI to be predicted is compared to the probability of each pixel point belonging to each category with a label, and the segmented WFSI is automatically generated. The same area per unit pixel is represented in a digital image, using pixel statistics to achieve WFP measurements. The ratio between the number of pixels in the whole image and the total number of pixels in the failure areas is the ratio between the total number of pixels in the failure areas and the total number of pixels in the sample image. The WFP is then calculated as follows:

(1) $$M=\frac{p\sum {{}_{\left(x,y\right)}}_{\in A}1}{p\sum {{}_{\left(x,y\right)}}_{\in =S}1}\times 100\%=\frac{\sum {{}_{\left(x,y\right)}}_{\in A}1}{\sum {{}_{\left(x,y\right)}}_{\in =S}1}\times 100\%$$

where M is WFP (%), p is Pixel areas per unit, $\sum {{}_{\left(x,y\right)}}_{\in A}1$ denotes the number of pixels in the wood failure areas of the image, $\sum {{}_{\left(x,y\right)}}_{\in =S}1$ is the number of pixels in the total areas of the specimen sample, in which, the ratio can be found.

#### 2.3.1. UNet Model

**Figure 5:**
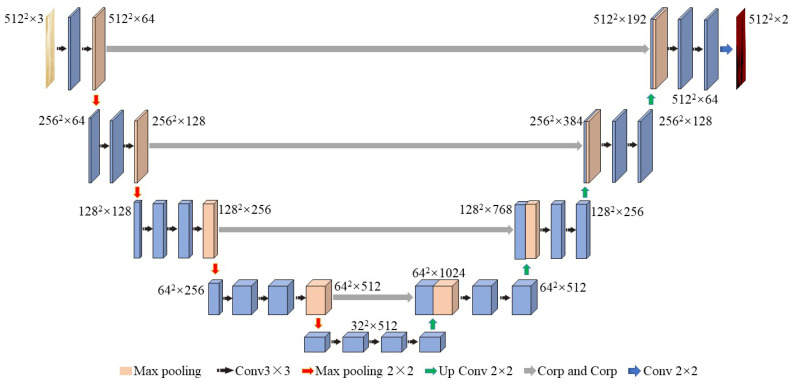
UNet structure diagram.

**Figure 6:**
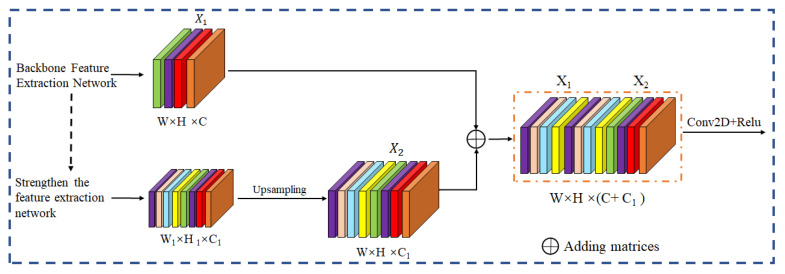
Feature extraction schematic.

UNet obtains accurate segmentation results by training a small amount of data [26,27]. The semantic segmentation of WFSI is carried out by taking advantage of the DL hierarchical representation. The UNet neural network used in this paper is divided into three main parts. The backbone feature extraction part uses a network of VGG16, consisting of convolution and maximum pooling to facilitate pretrained weights on the ImagNet, as shown in Figure 5, where five preliminary effective feature layers are available for the backbone feature extraction part [28,29]. The UNet enhanced feature extraction network is a U shape. The enhanced feature extraction network performs the feature fusion using the above five preliminary effective features by up-sampling and stacking the feature layers. To facilitate the construction of the network and for better generality, the UNet neural network is improved by directly up-sampling twice (Figure 6) as many layers before fusing them so that the feature layers obtained are of the same height and width as the input WFSI. 

#### 2.3.2. PSPNet Model

**Figure 7:**
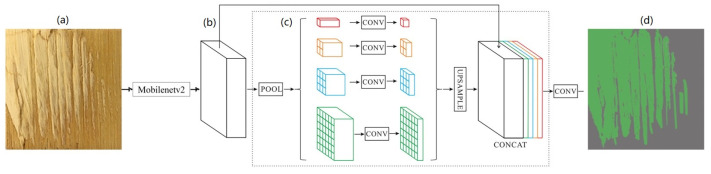
PSPNet model: (**a**) Input image; (**b**) Feature map; (**c**) Pyramid pooling module; (**d**) Final prediction.

PSPNet uses the pyramid pooling module to fuse semantic information at different scales, thus improving the ability of the model to obtain a global picture [30]. Compared with the UNet model, the PSPNet model is characterized by the use of a PSP module (Figure 7). The model proposes a pyramid pooling module that aggregates contextual information from different regions to improve the ability to obtain global information. The PSP structure can divide the acquired feature layers into grids of different sizes and each grid region is pooled equally within itself. This average pooling is beneficial to increase the receptive field of the separated layers, which is more intuitive and has a wider field of view.

#### 2.3.3. DeepLab_v3+ Model

**Figure 8:**
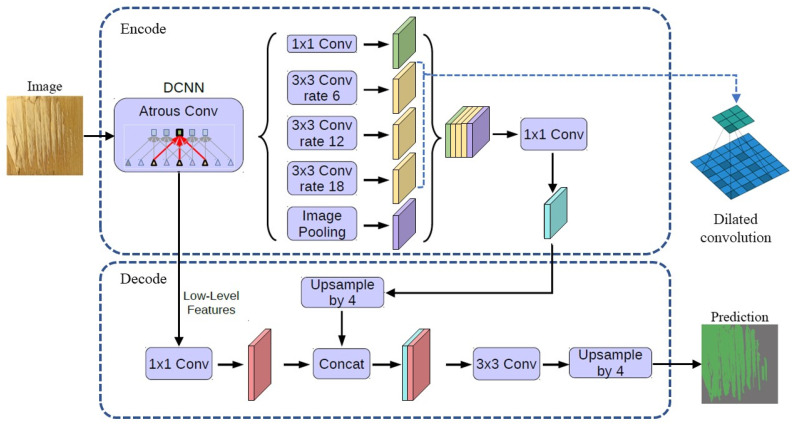
DeepLab_v3+ model.

The DeepLab_v3+ model (Figure 8) introduces the dilated convolution in the encoder part at the same time, combines the dilated convolution with the depth-separated convolution, and proposes the spatial pyramid pooling module while improving the receptive field [31]. The spatial pyramid pooling module is proposed to use a large number of convolutions with different dilated rates to further extract feature information at different scales for fusion. In the decoder part, unlike V3, which directly uses bilinear interpolation to the original image size, the feature map obtained from the encoder is first up-sampled four times, stitched with the feature map of the same size in the encoder, and then restored to the original size. Thus, DeepLab_v3+ achieves good results in different semantic segmentations [32].

### 2.4. Methods Validation

#### 2.4.1. Experimental Environment

The experimental environment is based on the Pytorch framework and the Python language, written in Pytorch 3.7.13 under VS Code 1.72.0 and trained with NVIDIA Geforce RTX3060 GPU.

#### 2.4.2. Model Parameters

After completing the data set preparation, experimental environment configuration, and algorithm model construction, the parameters of each model were finally determined after several tuning parameters, as shown in Table 3.

#### 2.4.3. Model Training

The three segmentation models are trained on the dataset established in this paper with 200 iterations after adjusting and setting the network parameters according to Table 2. Meanwhile, the performance of the model at the end of training is tested using the test set. According to the trend of the loss value of the validation set, we can judge whether the semantic segmentation model converges or not, and the size of the loss value is an important indicator to judge whether the model converges or not, and also the distance between the response label image and the predicted image, which is customized. However, the loss value is not 0 as the optimal indicator, but the model convergence is indicated when the loss value has a smooth trend.

#### 2.4.4. Evaluation Index of Semantic Segmentation Model

The indexes commonly used to evaluate the segmentation performance of semantic segmentation models are Recall, Precision, mAP, F1 score, and MIoU (Mean of Intersection over Union) [33], which can be calculated by Equations (2)–(5).

(2) Recall=TPTP+FN’

(3) $$\mathrm{Pr}ecision=\frac{TP}{TP+FP’}$$

(4) F1=2×Recall×PrecisionRecall+Precision

(5) $$MIoU=\frac{1}{k+1}\sum_{i=o}^{k}\frac{TP_{i}}{TP_{i}+FP_{i}+FN_{i}}$$

*TP* is the number of true positives, *FP* is the number of false positives, and *FN*^′^ is the number of false negatives when predicting the *i*th category, and *k* represents the *k* categories predicted [34].

## 3. Results and Discussion

### 3.1. Analysis of Model Training

#### 3.1.1. Results of Model Training of WFSI

**Figure 9:**
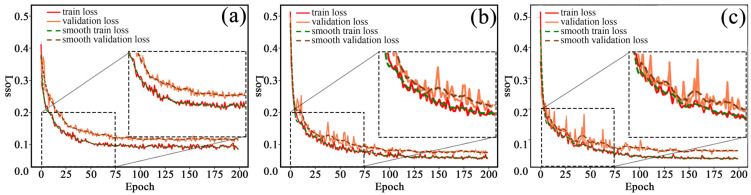
Different model training loss curves: (**a**) UNet model; (**b**) PSPNet model; (**c**) DeepLab*_*v3+ model.

Using the dataset in Section 2.2, under the experimental environment described in Section 2.4.1, the same experimental parameters were set to PSPNet, UNet, and DeepLab_v3+, respectively, and were then trained and tested. The variation of the train and validation loss values are shown in Figure 9. The performance of the model is analyzed based on the fit and convergence of the model. 

As shown in Figure 9, after the three models are trained on the same dataset, the change of validation loss curve is quite different. However, the three models show good fit. First, the validation loss curve tends to a steady state when the number of iterations of the UNet model (Figure 9a) exceeds 75, and the model basically converges. Second, the PSPNet model (Figure 9b) has a larger amplitude of the validation loss curve when the number of iterations is 0*–*75, and the model does not start to converge until the number of iterations exceeds 100. Finally, the validation loss curve fluctuation of DeepLab_v3+ model (Figure 9c) is larger compared with UNet and PSPNet when the number of iterations is between 0 and 100, and the trend of the validation loss is in a stable state only after 125 iterations until the end of 200 training sessions. Although, when examining the stability of loss values, the trend of change, and the convergence of the model, it can be seen that the UNet model is more advantageous. However, from the difference between the training set and the validation set and the degree of loss value decline, it can be seen that the advantages of the DeepLab_v3+ model are more prominent, the generalization ability is greater, and the train loss curve is closer to the validation loss curve. Because the deeplabv3+ model introduces a large dilated convolution in order to increase the perceptual field without using a pooling operation to increase the Receptive Field, the convergence is faster.

#### 3.1.2. Results of Semantic Segmentation of WFSI

Using the dataset in Section 2.2, under the experimental environment described in Section 2.4.1, the same experimental parameters were set to PSPNet, U-Net, and DeepLab_v3+, respectively, and were then trained and tested. The test results are shown in Table 3. According to the evaluation indexes of the semantic segmentation model described in Section 2.4.4, the test results are compared and analyzed to obtain the model with the best segmentation performance for the WFSI. The evaluation results of each model are shown in Table 4, and all three models achieved an accuracy rate of 94% or higher. Compared with PSPNet and DeepLab_v3+, the UNet model is the most effective and faster in wood failure area segmentation with 98.20% mPA, 98.96% recall, 96.83% precision, and 97.13% accuracy. However, in order to evaluate the models with superiority and inferiority in terms of synthesis, the F1 value is also usually used as an evaluation index. Among the three models, the F1 value of the UNet model is also greater than that of PSPNet and DeepLab_v3+, with a maximum improvement of 4.1%. The higher their index values are, the better the image segmentation effect of the model, the better the post-processing effect of the semantic segmented image, and the higher the calculation accuracy of picking points [35]. Therefore, UNet is selected as the semantic segmentation model of the WFSI of the WFP in this paper.

#### 3.1.3. Analysis of Model Prediction Results

**Figure 10:**
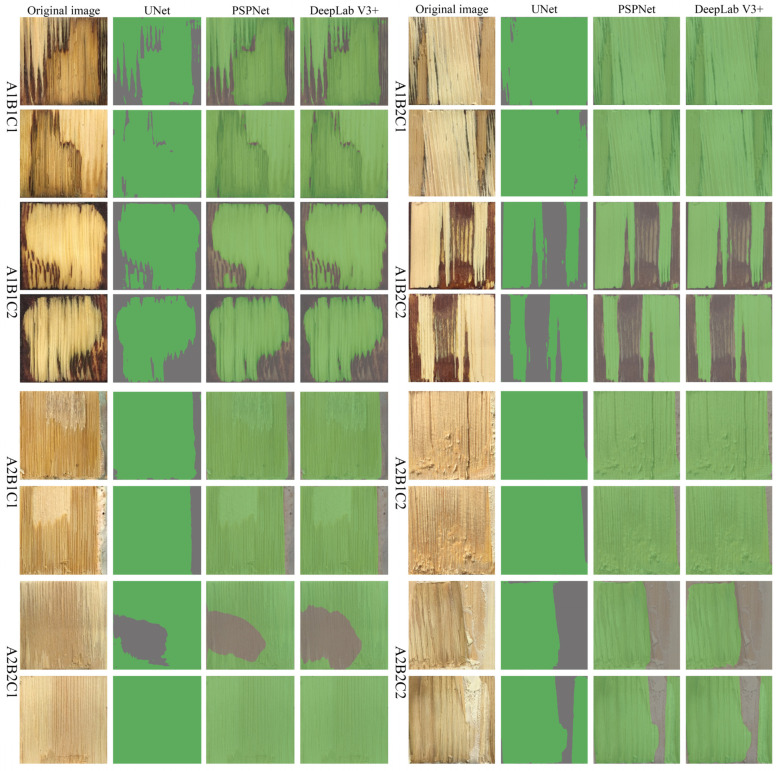
Prediction results of three semantic segmentation models under different conditions.

As can be seen from Figure 10, the three models can predict the wood failure areas under each test condition. However, due to the influence of the test conditions, the prediction results under different conditions differed significantly.

**Figure 11:**
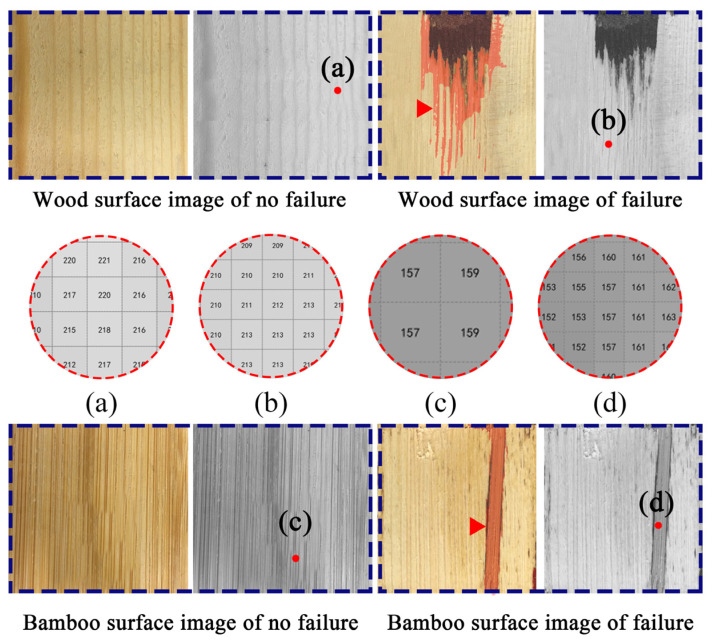
Local pixel value changes before and after failure of the base material: (**a**) Natural larch; (**b**) Larch after failure; (**c**) Natural carbonized bamboo; (**d**) Carbonized bamboo after failure.WOM.

First, basic materials variation had the greatest effect on WFP measurements. Wood-failure areas on the bamboo failure surface could be predicted in the A2B2C1 condition, but wood failure regions on the wood failure surface could not be predicted. This is due to the small difference in pixel values between wood-failure and unfailure wood regions (Figure 11) and the very similar semantic features, resulting in the inability of the model to segment this type of confusing semantic information. The three network models in the A2B1C1 condition performed better in distinguishing between the wood-failure regions where the object was a darker-colored adhesive. However, it was still not possible to distinguish the wood-failure areas on the wood-failure surface for prediction.

**Figure 12:**
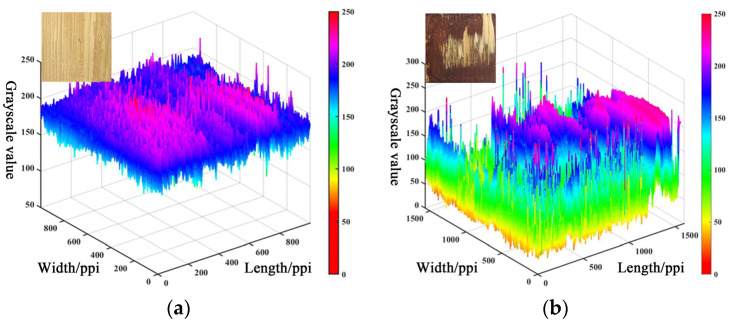
Effect of adhesive type change on grayscale value: (**a**) Effect of PF adhesives on grayscale values; (**b**) Effect of PF adhesives on grayscale values.

Second, the greater impact on the measurement is the adhesive color change. When the adhesive is PF, the WFP can be measured, and the difference with the actual wood failure areas is small (Figure 12a). However, when the adhesive is MDI, the prediction effect is relatively poor (Figure 12b) [36]. The main reason is that the inherent color of PF is darker compared with MDI, the inherent color difference with the wood failure areas is more obvious, and the semantic information is very clear [37]. Therefore, the PF adhesive region is more easily predicted.

Finally, it is the shear conditions that have an impact on the measurement results. The adhesive failure areas under wet conditions can be detected more accurately, but part of the adhesive layer failure areas under dry shear conditions is not predicted and, therefore, is identified as a wood failure area, causing measurement error. This is because under wet shear conditions, the inherent color of wood and adhesive deepens, and the difference in gray value are obvious, making the prediction of the wood failure areas more favorable.

Therefore, we can see from the prediction results that all three network models can achieve WFP measurement when the wood failure areas are large (the number of pixels is greater than 230) and relatively intact. However, when the wood failure areas are small (the number of pixels is less than or equal to 230) and relatively independent, the UNet model shows high accuracy, adaptability, and robustness. 

#### 3.1.4. Mechanisms for Predicting Wood Failure Surfaces

Semantic segmentation is mainly based on two elements of information, which are semantic information and spatial relationship features [38]. However, due to the different network structures of each model, it is impossible to obtain the same amount of information when obtaining both kinds of information. It is usually shown that when the semantic information is strong, the spatial relationship features are weaker; conversely when the spatial relationship features are larger, the semantic information is weaker. This is because the segmentation model reduces the dimensionality of the feature map by convolutional and pooling layers, which makes the features more advanced and semantic information richer. However, a large number of spatial relational features are lost. On the contrary, without the down-sampling layer, the feature map keeps the same size as the original image and maintains more spatial location information. However, the semantic information becomes poorer and more computationally intensive, but this spatial information is also crucial for accurate segmentation.

In the WFSI, there are only the wood failure and background regions, and the pixel points of the wood failure areas are no longer classified at the secondary level. Thus, the wood failure areas are not an instance segmentation, but a semantic segmentation, which focuses more on semantic information. The segmentation effect of semantic segmentation is strongly related to the receptive field of the model structure. First, the DeepLab_v3+ model improves the receptive field of the model by introducing dilated convolution, and PSPNet improves the receptive field of the model by averaging pooling over different levels. However, both receptive fields extract semantic information for a preliminary feature layer and cannot obtain deeper semantic information. Meanwhile, the semantic information of WFSI is simple and does not need to consider much contextual information, and both deep and shallow features are significant. Finally, due to the small dataset of WFSI and the complexity of preparing specimens, overfitting is prone to occur if large models such as DeepLab_v3+ and PSPNet are used. Therefore, it can be concluded from the model structure and characteristics, etc. that both DeepLab_v3+ and PSPNet models are not suitable for the segmentation of wood failure areas. The UNet model, on the other hand, achieves the purpose of increasing the receptive field by down-sampling and no longer divides the feature maps into shallow and deep feature maps. Secondly, the feature map obtained by deconvolution causes the missing edge features, and the missing features are supplemented by the skip-connection structure, which preserves the semantic information at each level, the segmentation effect is superior to the other two models, and it works best in wood-failure areas segmentation. Therefore, the UNet model is used as the model of our method in this paper.

### 3.2. Performance Evaluation of the Proposed Algorithm for WFP Measurement

#### 3.2.1. Measurement Accuracy

The measurement results are an important basis for measuring the feasibility of the measurement method, to fully verify the feasibility of our method. In this paper, based on the above test conditions, the same number of images of the failure surface of each specimen from the validation set were selected to measure the WFP by our method and the manual method, with a total of eight groups of six specimens each. ANOVA and error analysis was performed on the actual and image values (Table 5) to verify the feasibility of the method in the actual measurement process.

The analysis of variance (Table 6) shows that there are different levels of significant differences between the measurements of the manual method and our method under different conditions. The A1B1C1, A1B1C2, A1B2C1, A1B2C2 and A2B1C1 conditions show no significant differences; the A2B1C2 and A2B2C1 conditions show significant differences; and the A2B2C2 condition shows highly significant effects.

The relative error and absolute error analysis show that the maximum absolute error and the maximum relative error under A1B1C1, A1B1C2, A1B2C1, and A1B2C2 conditions are only 3.8 and 5.3%. The maximum relative error and absolute error under A2B1C2, A2B2C, and A2B2C2 conditions are 26.9 and 54.4%, respectively. Usually, the relative error better reflects the credibility of the measurement, and the relative error needs to be less than 20% to meet the actual engineering requirements. Therefore, it can be concluded that the measurement error of the specimens prepared using PF is smaller and the measurement accuracy is higher, and the errors are less than 20%, which can be applied to the actual production. However, there are more differences in the measurement errors of the specimens prepared using MDI, and the WFP measurement is not possible under other conditions, except A2B1C1. Because the MDI adhesive is very similar to the intrinsic color of the material, the DL method cannot predict the WFSI.

In summary, the adaptability of our methods under different conditions has a large difference, and the accuracy of WFP measurement is affected by the change in adhesive type. Among them, the measurement accuracy was affected by both shear conditions and basic materials variation when the adhesive types were the same.

#### 3.2.2. Measurement Speed

Currently, as industrialized production becomes more and more intense, high efficiency and speed are important signs of industrialized production. To verify that this paper has a faster measurement speed, six images of wood failure surfaces are randomly selected in the verification set and measured by the manual method and our method to verify the advantage of our method in terms of measurement speed.

**Figure 13:**
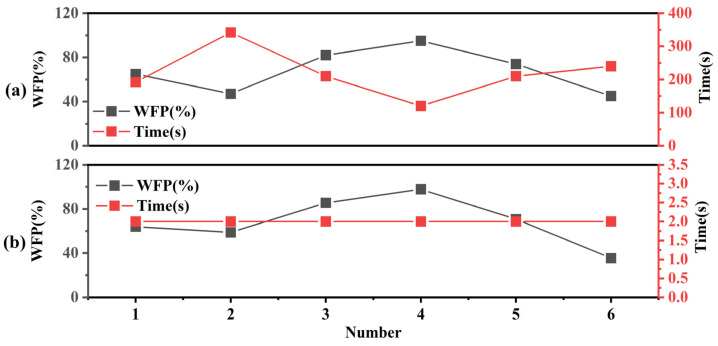
The relationship between measurement speed and WFP: (**a**) Manual method; (**b**) Our method.

As can be seen in Table 7, the two WFP measurement methods show a large difference in measurement time when the number of measured samples is the same. The manual method showed a large difference in measurement time for different sizes of WFP. When the WFP is 98%, it takes the least time, and when the WFP is 47%, the time can reach 342 s, and the average measurement time is 219 s. However, when the WFP is 82% and 74%, the measurement time is 210 s. On the contrary, the measurement time of our method is 2 s. Compared with the manual method, the measurement efficiency of our method is improved by 108.5 times, and it is not affected by the measurement size of the WFP (Figure 13b). At the same time, Figure 13 shows that the trend of measurement time and WFP is not consistent, which indicates that there is no large correlation between measurement speed and WFP.

**Figure 14:**
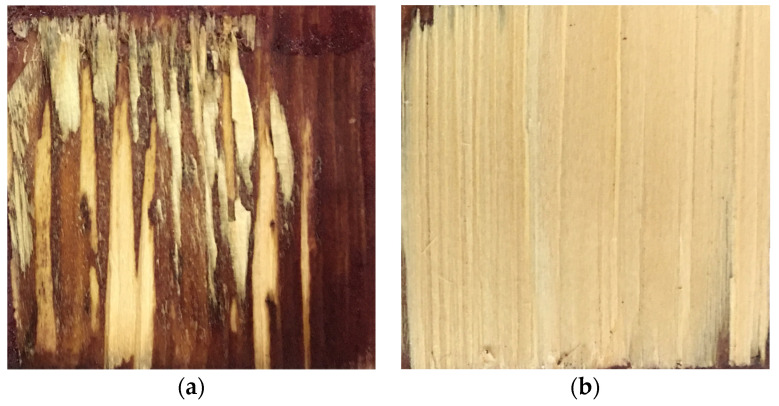
WFSI type. (**a**) Images that take a long time; (**b**) Images that take less time.

To explore the reasons for the large fluctuation of measurement time by manual methods (Figure 13b), this paper selected a typical case for analysis. It can be seen that the measurement time of Figure 14a is greater than that of Figure 14b. The wood failure areas of Figure 14a are more scattered, and the wood failure level is of different depths, so it takes a longer time for the tester to distinguish and confirm. Figure 14b shows that because the wood failure area belongs to the deep wood and is very concentrated, the WFP can be obtained by measuring only the smaller adhesive areas. It can be seen that the measurement speed is mainly affected by the complexity of the wood failure phenomenon, and the subjective dynamic factor is also one of the main factors affecting its cause.

#### 3.2.3. Measurement Stability

**Figure 15:**
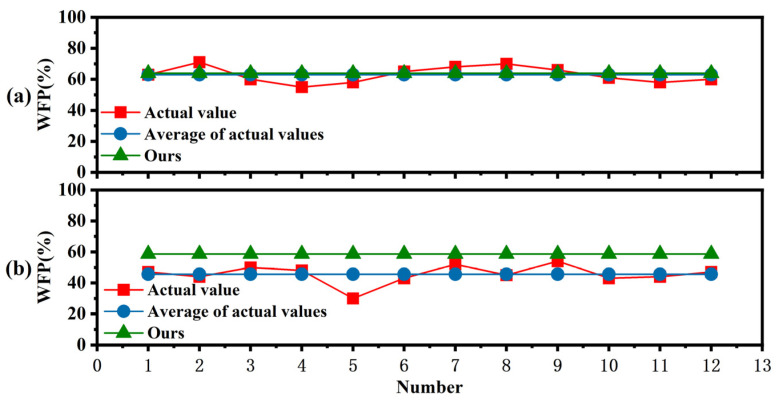
Stability analysis of both methods. (**a**) Time for 12 testers to measure the same sample; (**b**) One experimenter measures the time variation of 12 specimens.

The stability of the measurement method is an important element to measure measurement efficiency. In this paper, 12 trained experimenters were invited to measure the No. 1 specimens in Table 4 using two measurement methods, respectively. As can be seen in Figure 15a, the actual values hovered above and below their mean values with small amplitudes, but the maximum absolute error reached 8, with a coefficient of variation of 13.28%. However, our method is always consistent with the measured values under the variation of different experimenters. Next, one trained tester was invited to perform 12 measurements on the same specimen using both measurement methods, and the results are shown in Figure 15b. The measured values fluctuated above and below their mean values, but with a larger amplitude. Among them, the maximum measurement error with the measured values measured by the manual method and its mean value can reach 15.58, with a coefficient of variation of 8.1%. This is mainly because the manual method is affected by the subjective factors of the test personnel. When different testers measure the test only once, the attention is more focused, so the measurement error is smaller. However, when the same tester measures the same specimen 12 times, the testers’ attention decreases as the number of measurements increases and the measurement time increases. As the test procedure gradually becomes increasingly boring, the amplitude of the measurement results is larger. Our method requires only simple training, the coefficient of variation of wood failure measurement is 0, and the stability of measurement can reach 100%. Therefore, our method shows high stability and is a very scientific measurement method.

## 4. Conclusions

This paper presents a new method to accurately measure WFP based on DL. This study trains the same dataset using UNent, PSPNet, and DeepLab_v3+ models, and the results show that the UNet model has better convergence and fit than the PSPNet and DeepLab_v3+ models. Meanwhile, this paper uses the semantic segmentation model based on the UNet network structure to segment the WFSI. The Precision, mPA, MIoU, and F1 of wood failure areas segmented by the UNet model were 96.96%, 98.20%, 98.87%, and 97.88, respectively. Compared with the main stem segmentation model based on PSPNet and DeepLab_v3+, it has higher segmentation accuracy; therefore, UNet is the most promising network model for main stem detection segmentation.

To verify the feasibility of our method, the three aspects of measurement accuracy, measurement speed, and stability were compared with the manual method, respectively. The results show that our method can achieve WFP measurement under some conditions, there is no significant difference with the actual measured value, and the relative error is less than 20%. The measurement speed of our method is 108.5 times faster than that of the manual method. The maximum coefficient of variation of the manual method is 13.28%, but the coefficient of variation of our method is 0. This shows that our method can not only be applied to practical production but also has greater advantages in terms of speed and stability.

Although our method can measure WFP, the measurement accuracy is also affected by the test environment. The influence of each factor on the predicted results also has a size difference. In descending order, they are basic materials > adhesive > shear conditions. Additionally, it can be further subdivided into basic materials: carbonized bamboo > larch; adhesive: PF > MDI; shear condition: wet state > dry state.

## Figures and Tables

**Table 1:** Variation and experimental level of specimen.

Level Factor	Adhesive (A)	Shear State (B)	Base Material (C)
1	PF	Wet	Larch-bamboo
2	MDI	Dry	Larch-Larch

**Table 2:** Test combination of specimens.

Group	Code	Adhesive (A)	Shear State (B)	Base Material (C)
1	A1B1C1	PF	Wet	Larch-bamboo
2	A1B1C2	PF	Wet	Larch- Larch
3	A1B2C1	PF	Dry	Larch-bamboo
4	A1B2C2	PF	Dry	Larch- Larch
5	A2B1C1	MDI	Wet	Larch-bamboo
6	A2B1C2	MDI	Wet	Larch- Larch
7	A2B2C1	MDI	Dry	Larch-bamboo
8	A2B2C2	MDI	Dry	Larch- Larch

**Table 3:** Parameter setting of the model.

Model Name	Backbone Feature Extraction Network	Image Size	Bulk	Learning Rate	Momentum	Attenuation
Unet	vgg16	512 × 512	2	1 × 10^−5^	*	*
Pspnet	mobilenetv2	512 × 512	5	1 × 10^−2^	0.9	1 × 10^−4^
DeepLab_v3+	mobilenetv2	512 × 512	2	7 × 10^−3^	0.9	1 × 10^−4^

* means “no data”.

**Table 4:** The segmentation performance of wood failure areas under different models.

Model	mPA/%	Recall/%	Precision/%	MIoU/%	Accuracy/%	F1
Unet	98.20	98.96	96.83	98.87	97.13	97.88
Pspnet	96.67	96.64	96.47	93.97	94.25	96.55
DeepLab_v3+	95.48	93.47	94.58	95.21	94.83	94.02

**Table 5:** WFP measurement results.

Code	Method	WFP/%					
		No. 1	No. 2	No. 3	No. 4	No. 5	No. 6
A1B1C1	manual	55	40	66	70	76	30
	Our	56.4	41.8	70.9	72.2	79.5	32.3
A1B1C2	manual	40	75	60	85	70	75
	Our	44.1	76.3	61.6	88.8	75.0	76.4
A1B2C1	manual	80	85	90	75	82	95
	Our	85.5	89.3	92.4	77.7	79.0	91.1
A1B2C2	manual	70	80	85	55	75	80
	Our	74.3	82.4	90.2	59.6	80.1	78.7
A2B1C1	manual	35	42	60	42	71	55
	Our	42.2	50.8	66.8	50.0	60.5	65.4
A2B1C2	manual	75	82	65	28	64	53
	Our	90.7	100	86.9	63.7	96.6	90.9
A2B2C1	manual	88	65	80	78	90	50
	Our	94.1	87.4	93.5	89.5	98.7	88.8
A2B2C2	manual	77	85	93	84	55	76
	Our	100	97.5	100	99.5	95.4	96.9

**Table 6:** ANOVA and error analysis of WFP.

Code	ANOVA F-Value	ANOVA *p*-Value	ANOVA F-Crit	ANOVA Significance	Absolute Error	Relative Error (%)
A1B1C1	0.064	0.805	4.964	a	2.7	4.9
A1B1C2	0.101	0.757	4.964	a	2.9	4.7
A1B2C1	0.118	0.738	4.964	a	3.6	4.3
A1B2C2	0.313	0.588	4.964	a	3.8	5.3
A2B1C1	0.564	0.471	4.964	a	8.6	17.6
A2B1C2	8.263	0.016	4.964	b	26.9	54.4
A2B2C1	6.844	0.025	4.964	b	16.8	26.7
A2B2C2	13.767	0.004	4.965	c	19.9	28.6

Note: *p* value > 0.05 is not significant (a); *p* value ≤ 0.05 is significant (b); *p* value ≤ 0.01 is very significant (c).

**Table 7:** Measurement time for both methods.

Number	Manual Method		Our	
	WFP/%	Time/s	WFP/%	Time/s
No. 1	65	192	63.9	2
No. 2	47	342	58.7	2
No. 3	82	210	85.5	2
No. 4	98	120	97.8	2
No. 5	74	210	70.7	2
No. 6	45	240	35.4	2

## Data Availability

Not applicable.
